# Identification of antiparasitic drug targets using a multi-omics workflow in the acanthocephalan model

**DOI:** 10.1186/s12864-022-08882-1

**Published:** 2022-09-30

**Authors:** Hanno Schmidt, Katharina Mauer, Manuel Glaser, Bahram Sayyaf Dezfuli, Sören Lukas Hellmann, Ana Lúcia Silva Gomes, Falk Butter, Rebecca C. Wade, Thomas Hankeln, Holger Herlyn

**Affiliations:** 1 Institute of Organismic and Molecular Evolution (iomE), Anthropology, Johannes Gutenberg University Mainz, Mainz, Germany; 2 Present address: Institute for Virology, University Medical Center of the Johannes Gutenberg University Mainz, Mainz, Germany; 3 Molecular and Cellular Modeling, Heidelberg Institute for Theoretical Studies, Heidelberg, Germany; 4 Department of Biology and Evolution, University of Ferrara, Ferrara, Italy; 5 Institute of Organismic and Molecular Evolution (iomE), Molecular Genetics and Genomic Analysis, Johannes Gutenberg University Mainz, Mainz, Germany; 6 Present address: Nucleic Acids Core Facility, Johannes Gutenberg University Mainz, Mainz, Germany; 7 Federal University of Amazonas, LAPAA, Manaus, Brazil; 8 Quantitative Proteomics, Institute of Molecular Biology (IMB), Mainz, Germany; 9 Center for Molecular Biology (ZMBH) and Interdisciplinary Center for Scientific Computing (IWR), Heidelberg University, Heidelberg, Germany

**Keywords:** Parasites, Anthelmintics, Target molecule, Virtual ligand screening, Active ingredients, Medical genomics

## Abstract

**Background:**

With the expansion of animal production, parasitic helminths are gaining increasing economic importance. However, application of several established deworming agents can harm treated hosts and environment due to their low specificity. Furthermore, the number of parasite strains showing resistance is growing, while hardly any new anthelminthics are being developed. Here, we present a bioinformatics workflow designed to reduce the time and cost in the development of new strategies against parasites. The workflow includes quantitative transcriptomics and proteomics, 3D structure modeling, binding site prediction, and virtual ligand screening. Its use is demonstrated for Acanthocephala (thorny-headed worms) which are an emerging pest in fish aquaculture. We included three acanthocephalans (*Pomphorhynchus laevis, Neoechinorhynchus agilis*, *Neoechinorhynchus buttnerae*) from four fish species (common barbel, European eel, thinlip mullet, tambaqui).

**Results:**

The workflow led to eleven highly specific candidate targets in acanthocephalans. The candidate targets showed constant and elevated transcript abundances across definitive and accidental hosts, suggestive of constitutive expression and functional importance. Hence, the impairment of the corresponding proteins should enable specific and effective killing of acanthocephalans. Candidate targets were also highly abundant in the acanthocephalan body wall, through which these gutless parasites take up nutrients. Thus, the candidate targets are likely to be accessible to compounds that are orally administered to fish. Virtual ligand screening led to ten compounds, of which five appeared to be especially promising according to ADMET, GHS, and RO5 criteria: tadalafil, pranazepide, piketoprofen, heliomycin, and the nematicide derquantel.

**Conclusions:**

The combination of genomics, transcriptomics, and proteomics led to a broadly applicable procedure for the cost- and time-saving identification of candidate target proteins in parasites. The ligands predicted to bind can now be further evaluated for their suitability in the control of acanthocephalans. The workflow has been deposited at the Galaxy workflow server under the URL tinyurl.com/yx72rda7.

**Supplementary Information:**

The online version contains supplementary material available at 10.1186/s12864-022-08882-1.

## Background

The global market for antiparasitics, or parasiticides, currently amounts to about seven billion euros per year [1]. Most of this is spent on meat production but the share of expenditure in the aquaculture of fish is very likely to significantly grow in near future. With about 50 million tons per year, the production of animal protein in aquaculture is now already of great importance for many countries. However, many of these follow the agenda to increase the aquaculture branch, for securing high-quality food supply to their often growing populations [2]. As a result, revenues in fish aquaculture, currently estimated at around 140 billion dollars per year, will also rise [3], as will the losses due to diseases of yearly around 6 billion US dollars [4]. Accordingly, the control of parasites of taxa such as Nematoda (roundworms), Platyhelminthes (flatworms), and Acanthocephala (thorny-headed worms) in fish aquaculture is a major issue.

Extracts from garlic [5], thyme [6] and different species of the Fabaceae genus *Copaifera* [7] might have potential in the control of Acanthocephala (reviewed in [8]). However, to date no reliable agent has passed clinical evaluation. In addition to phytoextracts, a whole arsenal of chemical anthelmintics is available [9, 10]. Although effective, a widespread disadvantage is a low specificity as reflected in the use against a broad range of taxa (survey e.g. in [11]). Well-known examples are niclosamide and benzimidazole derivates, which are used against phylogenetically distant parasites [9, 12, 13]. But snails, unicellular species, invertebrate metazoans, and algae can be negatively affected as well [12, 14, 15]. Their pro-apoptotic activity in the broadest sense even confers potential on diverse anthelmintics as cytostatic agents in cancer therapy [16–24]. Consequently, when dissipated into surrounding waters, deworming agents and their mostly under-investigated metabolites might cause harm to a broad range of species (e.g., [25]). Also, detrimental long-term effects appear possible since anthelmintic metabolites can accumulate and persist in the environment [26, 27]. Additional concerns arise from the growing number of resistant parasite strains (e.g., [28–30]) while almost no new anthelmintics are being developed [31, 32]. Thus, there is a need for the development of novel strategies in parasite control.

A time- and cost-saving approach lies in the mechanism-based screening of compound libraries for ligands to parasitic target molecules. An important point here is that advances in genomics, transcriptomics, and proteomics target now enable the determination of functionally highly important and specific target molecules in parasites. Favorable for ligand-screening is further the recent leap in protein structure prediction. Indeed, with the recent progress in 3D structure modelling (AlphaFold2) it is now possible to predict 3D models with much greater precision de novo than before [31]. Nevertheless, traditional methods were prevailing in the development of drugs against parasites until recently [32–34], with comparably few exceptions so far. For example, several studies used omics for identifying drug targets in viruses (e.g., SARS-CoV-2 [35]), malaria-causing *Plasmodium falciparum* [36], and additional unicellular pathogens [37]. In addition, several potential antigens have been identified for nematode and platyhelminth parasites of humans, lifestock, and pets, including *Ancylostoma duodenale*, *Ascaris lumbricoides*, *Brugia malayi*, *Echinococcus granulosus*, *Fasciola hepatica*, *Haemonchus contortus*, *Necator americanus, Onchocerca volvulus*, *Ostertagia ostertagi*, *Schistosoma* spp., *Strongyloides stercoralis*, *Taenia solium*, *Teladorsagia circumcincta*, *Toxocara canis*, and *Trichuris trichiura* (reviewed in [38]). These examples also reflect the increasing power of omics-guided antigen or target identification, with genomics providing the basis for transcriptomics and proteomics. Consequently, recently proposed strategies in the field increasingly rely on proteomics or a combination of genomics, transcriptomics, and proteomics. Corresponding workflows inherently integrate gene annotations and ontologies [38, 39]. But to the best of our knowledge, there is yet no bioinformatics workflow integrating various omics techniques, annotation, gene ontology analysis, 3D modelling, and virtual ligand screening into the development of novel strategies in parasite control. Here we present a corresponding workflow for the determination of candidate target proteins, the disruption or blocking of which should effectively and specifically kill parasites. We preliminarily characterize the parasite proteins focused, model their 3D structure, and present potential ligands with known properties. To demonstrate its general applicability, we establish the procedure in acanthocephalans.

Acanthocephalans are common parasites in the intestinal tract of many mammals, amphibians, birds, turtles, lizards, snakes, and fishes (e.g., [40]). Depending on the host and the intensity of infection, the worms might penetrate the intestinal wall, which can cause fatal peritonitis [41]. Migrating worms also damage other host organs and mesenteries [42]. Inside the intestine, acanthocephalans injure the intestinal wall with their usually hook-bearing attachment organ, the proboscis [43–45]. The resulting lesions reduce the absorptive surface and hence lower the ability of the host to take up nutrients [43]. The gutless worms also absorb minerals and nutrients via their tegument, which they withhold or withdraw from the host [46–48]. Intestinal obstruction can also be fatal due to mass infections with up to ~ 1500 thorny-headed worms per individual host in the wild (e.g., [49, 50]). Acanthocephalans additionally parasitize human livestock, including domestic pig (*Sus scrofa domestica*) and chicken (*Gallus gallus domesticus*) (e.g., [47, 51]). They are also regular members of the parasitic fauna in marine fish aquaculture (e.g., [11, 52, 53]). Infections with acanthocephalans are further documented for limnocultures of brown trout (*Salmo trutta fario*), pirarucu (*Arapaima gigas*), Nile tilapia (*Oreochromis niloticus*), and tambaqui (*Colossoma macropum*), amongst others [54–57]. Here, high intensities can cause reduced growth, weakening, and emaciation of the fish. Deformations and death of infected fish have also been reported [52, 54, 58]. In Brazil, for instance, acanthocephaliasis is regarded as the main obstacle to successful aquaculture [8, 45, 59–64].

First genome and transcriptome assemblies for Acanthocephala have lately been published for the Eurasian species *Pomphorhynchus laevis* (Zoega in Müller, 1776) Monticelli, 1905 (Acanthocephala: Palaeacanthocephala) [65]. In the present investigation, we included worms from common barbel (*Barbus barbus*), a definitive host in which *P. laevis* matures and reproduces [66, 67], and European eel (*Anguilla anguilla*), in which *P. laevis* survives [68] but usually does not mature and reproduce [69]. For raising effectiveness of any yet-to-be determined agent against acanthocephalans, we searched for transcripts with little variation in abundance at a high level in 20 male and female *P. laevis* specimens from barbel and eel. To enable enhanced specificity of a future control of acanthocephalans, we screened for transcripts which were unique to or at least highly derived in *P. laevis* compared to diverse non-acanthocephalan species. Moreover, the ideal candidate target had to be readily accessible to drugs. This criterion was approximated by searching proteome data of *P. laevis* body walls for high-abundance proteins. To increase transferability of the results, protein sequences were checked for matches in new draft genomes of two additional fish-parasitizing species (Acanthocephala: Eoacanthocephala). These were *Neoechinorhynchus agilis* (Rudolphi, 1819) Van Cleave, 1916 from Adriatic thinlip mullet (*Chelon ramada*) and *Neoechinorhynchus buttnerae* Golvan, 1956 parasitizing above-mentioned tambaqui in South-American limnocultures. Predicted 3D models of the proteins meeting all these requirements were used to screen for drugs that might bind to acanthocephalan target proteins.

## Results

### Sequencing and mapping

Male and female specimens of *P. laevis* (Palaeacanthocephala) from two different host species were used for sequencing, resulting in four pairings of worm sex and host species. Each group included five worm specimens, so possible confounding factors in individual samples should not have affected downstream analysis. The resulting 20 RNA-Seq datasets contained 32.6 million single-end reads (75 bp) on average. Quality processing and mapping to an amended version of the reference transcriptome [65] was successfully passed by 95.1% of the sequences.

### Candidate target proteins for drug search

**Fig. 1:**
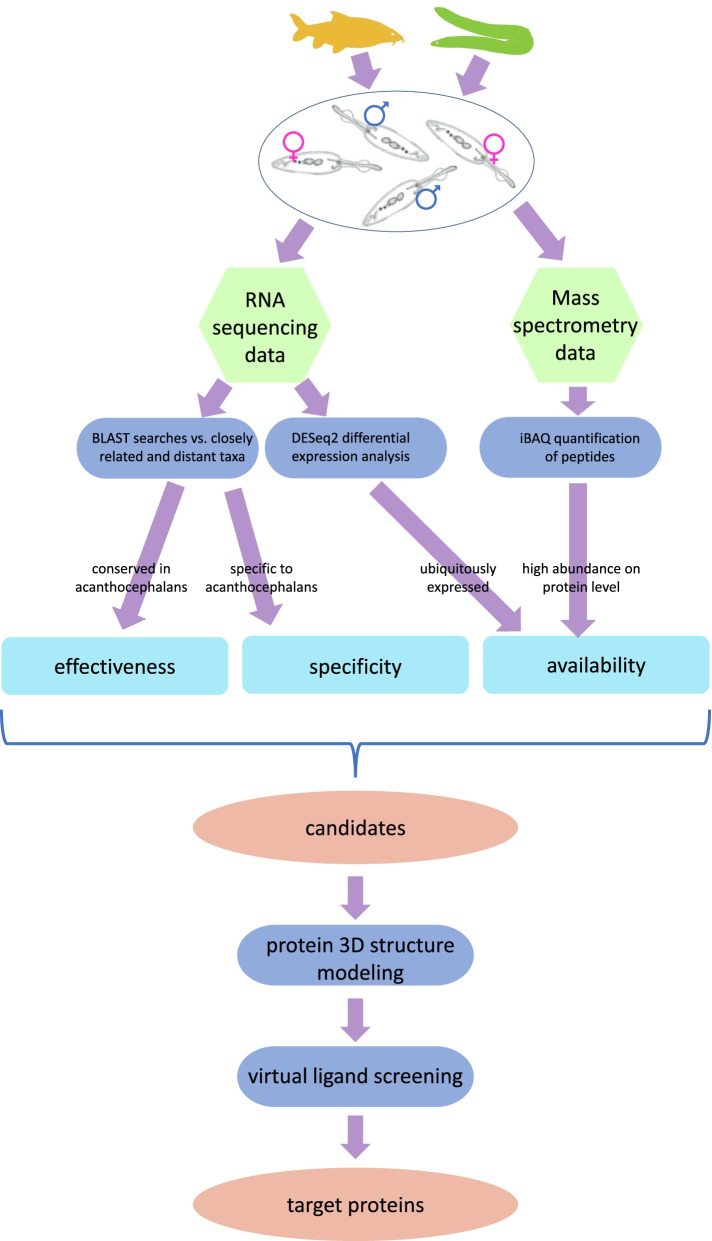
Flowchart of the analysis workflow. Female and male worm specimens were collected from two different hosts and used for mRNA sequencing and mass spectrometry. In-depth analyses ensured target identification (the target sequence is present in acanthocephalans), specificity (the target sequence is absent or has little sequence similarity in non-acanthocephalan species), and availability and accessibility (the target is present as protein in the acanthocephalan body wall). Candidate target protein sequences that fulfill these criteria were passed on to protein modeling and ligand screening

**Fig. 2:**
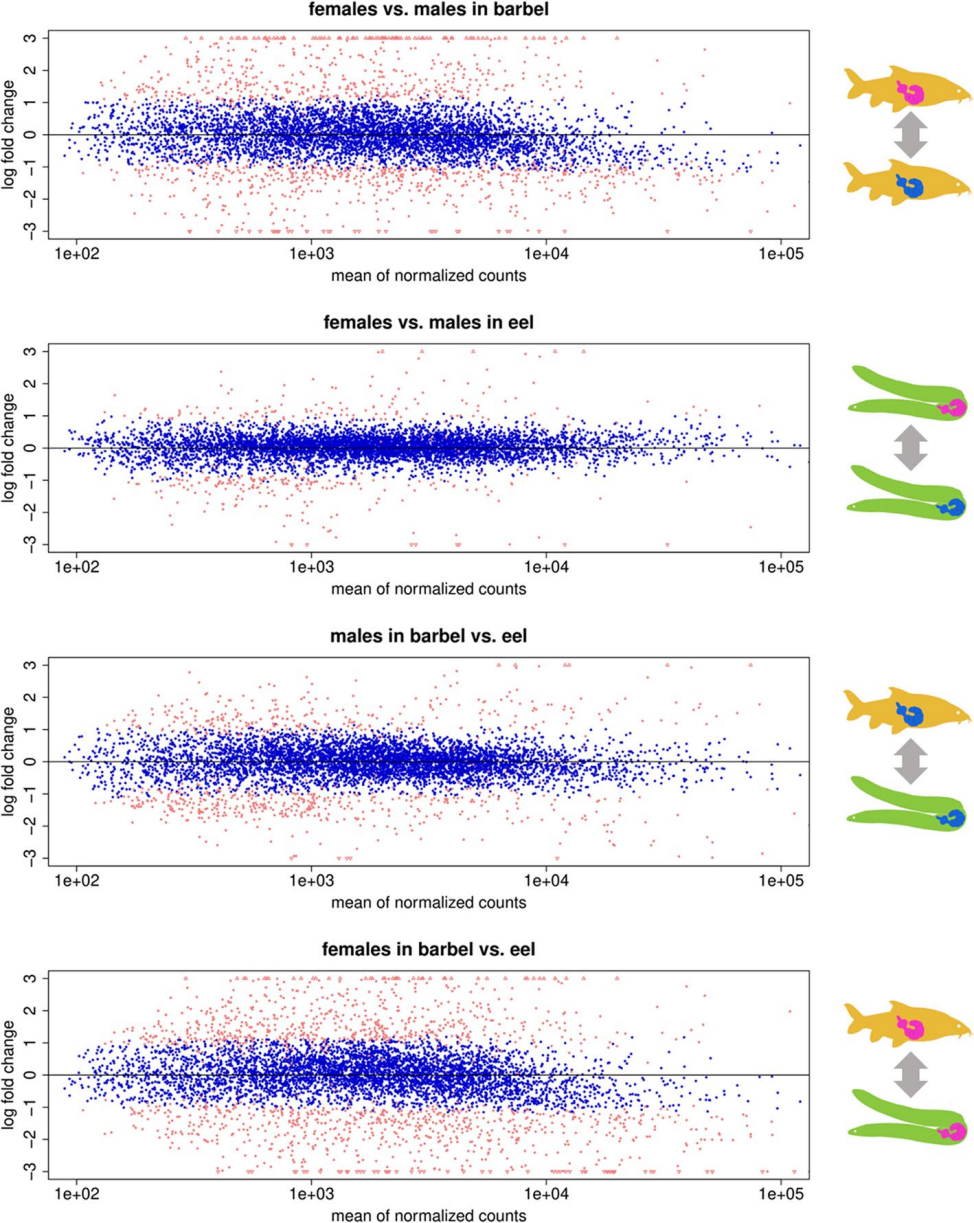
Analysis of transcript abundance. Shown are differential expression values for genes with at least 50 transcript reads in every sample. Each dot represents one gene. Genes with significantly similar transcript abundances (log fold change < 1.5; adjusted *p*-value < 0.05) are labeled blue, the remaining ones red. Only genes with similar transcript abundances in at least two of the four comparisons were kept for downstream analyses. Up- and downward pointing triangles at the top and bottom margins of the plots represent data points outside of the range depicted. Only a few isolated data points to the right have been omitted for better display

Mapping with RSEM revealed that the reads from RNA-Seq spread across transcripts of 18,740 genes. For downstream analysis of transcript abundance (DESeq2) we only considered genes that had at least 50 mapped transcript reads in each of the 20 samples, thus suggesting ubiquitous expression in *P. laevis* (availability criterion in Fig. 1; Supplementary Fig. S1). As an indication of low regulation up to constitutive expression, the log fold change of transcript abundance was set to ≤1.50 (adjusted *p*-value < 0.05) in a minimum of two out of four pairs of comparisons: (1) female vs. male worms from barbel, (2) female vs. male worms from eel, (3) male worms from barbel vs. eel, and (4) female worms from barbel vs. eel (Fig. 2; Supplementary Table S1). From the transcripts fulfilling this requirement, we extracted all open reading frames (ORFs) of at least 30 codons. Subsequent BLASTs for translated ORFs reduced the number of candidate targets to 121 (effectiveness & specificity criteria in Fig. 1). The corresponding *P. laevis* sequences had tblastn hits with E-values <1e-50 in novel genome assemblies of two fish-parasitizing acanthocephalan species from Eoacanthocephala, *N. buttnerae* and *N. agilis* (Supplementary Notes S1 & S2). Thus, drugs to be developed against them should be effective not only in *P. laevis* but fish-parasitizing acanthocephalans in general. The 121 candidates additionally lacked matches in six closer phylogenetic relatives from the Rotifera-Acanthocephala clade (Syndermata or Rotifera *sensu lato*) and in the SwissProt database at an E-value <1e-50 (tblastn/blastp). Correspondingly, agents tailored to these targets should specifically impair acanthocephalans but no other taxa.

**Fig. 3:**
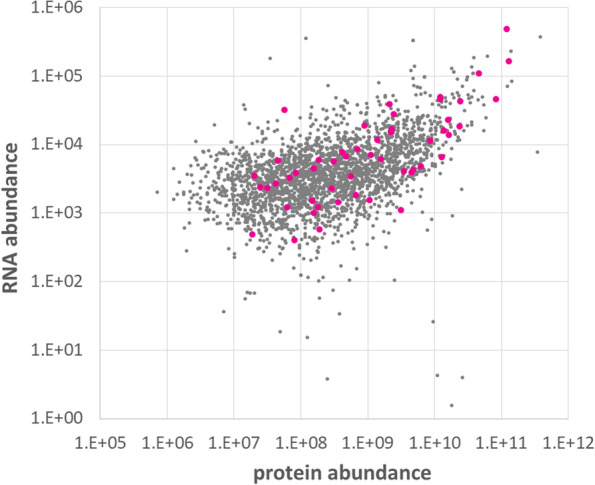
Correlation transcript and protein abundances. Each dot represents one of ~ 2500 proteins quantified by mass spectrometry. Candidate target proteins are highlighted in pink. Protein and transcript (RNA) abundances are given as iBAQ values and mean read counts, respectively. The correlation between the two abundances was moderately positive for all proteins (0.51; *p*-value = 2.4 e-165; Student’s *t*-test) and strongly positive for the candidate target proteins (0.81; *p*-value = 4.4 e-13; Student’s *t*-test). Given the levels of *p*-values, correction for multiple testing would not have affected the determination of significance

Since transcript abundance does not necessarily correlate with protein abundance (e.g., [70]), we validated the above results in proteome data. Corresponding mass spectrometry (MS) analysis focused on the acanthocephalan tissue promising easiest targetability, i.e., the body wall enclosing the gutless worms (availability criterion in Fig. 1). Based on five pools of *P. laevis* body-walls, the mass spectra matched 2548 ORFs in the reference transcriptome. Abundances of these proteins (iBAQ values) were significantly positively correlated with transcript abundances according to read counts (coefficient of correlation = 0.51, *p*-value = 2.4 e-165, Student’s *t*-test). Thus, higher transcript abundance overall indicated elevated protein abundance. The search for parasitic target molecules was continued with 52 MS-verified proteins (Fig. 3). As to be expected, transcript and protein abundances of these 52 target candidates were even more strongly correlated (coefficient of correlation = 0.81, *p*-value = 4.4 e-13, Student’s *t*-test).

The choice of candidates was supported by the fact that the matching rate between both subsets (52/121 = 0.43) was about three times higher than between the corresponding full lists (2548/18,740 = 0.14). In further support of the validity of the approach, the 52 proteins matched several expected properties. Thus, their mean length (485 amino acid residues) was very close to the average in eukaryotes (472 amino acid residues), and clearly exceeded corresponding averages in unicellular species (ca. 300 amino acid residues) [71]. Furthermore, the amino acid frequencies of the candidates retained was strongly positively correlated with previously reported average values across 614 eukaryotic proteomes (coefficient = 0.88, *p* = 3.6 e-07, Student’s *t*-test; Supplementary Table S2) [72]. Of the 52 candidate target proteins, 46 were characterized to be overall hydrophilic (88.5%), and eleven were predicted to have transmembrane helices (21.2%) (Supplementary Table S3). PFAM motifs were found in 46 of the candidate target proteins (88.5%), and Prosite motifs in 34 (65.4%) (Supplementary Table S4).

### Protein structure and binding site prediction

The above filtering for dissimilar genes ruled out to use structure models of homologous proteins in non-acanthocephalan species as a starting point. In fact, a database search (NCBI) did not reveal a deposited 3D model of a protein structure for any of the 52 candidate target proteins in *P. laevis*. This prompted us to perform de novo predictions using AlphaFold2 [31], which in 44 cases succeeded in providing a 3D model of the protein structure. AlphaFold2’s per-residue confidence metric pLDDT (range: 0–100) was 75.7 averaged across all candidates, with mean values ranging from 41.7 to 95.3 for the individual proteins. For estimating the precision of the structure predictions, we employed an additional protein structure prediction program, RoseTTAFold [73] (Supplementary Table S5). Comparison using the Dali Protein Structure Comparison Server [74] revealed high similarity of the models predicted. Thus, Dali’s average confidence or z-score for model comparison was 24.2, which is far beyond the threshold of strong matches (> 2). Likewise, Dali rated the average identity between models from AlphaFold2 and RoseTTAFold predictions as 85.5%, which considerably exceeds the threshold of significant similarities (> 20% [75];).

### Virtual ligand screening

**Fig. 4:**
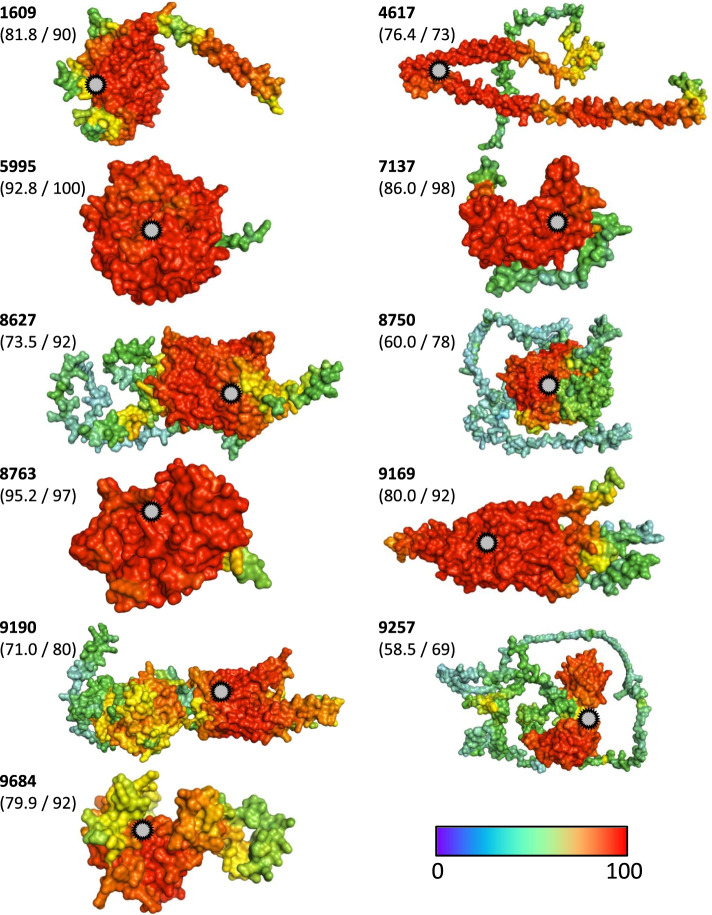
Three-dimensional structure models of the eleven top candidate target proteins. Shown are de novo models of 3D structures (constructed using AlphaFold2) for eleven proteins which fulfilled all filter criteria. The proteins were each additionally predicted to bind a drug with a free energy of ≤ −9.0 kcal/mol in the virtual screening using AutoDock Vina. The proteins are shown as molecular surfaces colored by AlphaFold2 confidence score (pLDDT; with higher values having greater confidence). Gray markings indicate predicted binding sites (on the surface or within the protein). Values in parentheses below protein identifiers give the average pLDDT of the protein model followed by the percentage identity between this model and one from a second 3D structure prediction program, RoseTTAFold. Both values are on the scale 0–100

Based on the AlphaFold2 models, COACH-D [76] identified putative ligand binding sites in each of the remaining 44 target candidates. In two of the protein models, a secondary binding site was predicted. Although confidence (c) scores varied widely (0.04 to 0.88), we retained all putative binding sites for ligand screening. For each of the 44 candidate targets, virtual screening of clinically tested and approved compounds using AutoDock Vina [77] identified ligands. Supplementary Table S6 provides previous knowledge on indications and molecular targets of these ligands, as extracted from various databases (ChEMBL, ClinicalTrials.gov, DrugBank, PubChem). Applying an arbitrary threshold of binding energy (− 9 kcal/mol), ten compounds remained as strongest-binding ligands to eleven candidate targets (Tables 1, 2). The discrepancy in number reflected that two of the acanthocephalan proteins shared tadalafil as strongest-binding ligand (Fig. 4; Table 1). It is further worthwhile noting that one of the ligands, the nematocidal anthelmintic derquantel, was predicted to bind second strongest to the model of protein 1609, in addition to its strongest binding to protein model 4617. The other eight ligands were each predicted to dock strongly to single acanthocephalan targets only (Table 2).

**Table 1:** Candidate target proteins in acanthocephalans and known drugs predicted to bind to them

Protein identifier	PFAM motif	Subcellular localization	Ligand (Drugs-lib in MTiOpenScreen [78])	Binding energy (kcal/mol)
1609^#^	glycosyl transferase	Golgi apparatus, membrane	Pranazepide (Derquantel)	−10.9
4617	troponin	nucleus, soluble	Derquantel	−9.0
5995^#^	amine lyase	cytoplasm, soluble	Tadalafil	−9.0
7137^#^	protein kinase	nucleus, soluble	Casopitant	−10.8
8627	unknown function	cell membrane, membrane	Afacifenacin	−11.4
8750^#^	PIP5K	cytoplasm, soluble	Piketoprofen	−9.9
8763^#^	NAD binding	peroxisome, soluble	Bemcentinib	−11.8
9169^#^	phosphatidic acid phosphatase	cell membrane, membrane	Tadalafil	−9.2
9190^#^	dopamine beta-monooxygenase	cell membrane, membrane	Fluazuron	−12.9
9257	RNA recognition motif	nucleus, soluble	Heliomycin	−9.1
9684^#^	–	cytoplasm, soluble	Etoposide	−9.4

Ligands predicted to bind strongest with a minimum free energy of −9 kcal/mol are shown. Parentheses give a case of second most strongly binding by derquantel. Hash signs mark proteins predicted to possess enzyme activity based on ECPred

*PFAM* Protein families

Standard InChI keys and 2D structures of the ligands are available in Supplementary Table S10

**Table 2:** Properties of selected ligands for assessing their drug-likeness

Agent	Molecular weight	nHA	nHD	nRot	nRing	MaxRing	nHet	fChar	nRig	Stereo centers	TPSA	logS	logP	logD	RO5
**Pranazepide**	438.45	6	2	4	6	12	7	0	33	1	77.56	−5.6	4.23	3.76	Yes
**Derquantel**	479.6	7	2	0	8	14	7	0	34	6	74.27	−4.97	4.4	2.6	Yes
**Tadalafil**	389.41	7	1	1	6	17	7	0	32	2	74.87	−5.36	2.9	2.43	Yes
**Casopitant**	616.62	6	0	9	4	6	13	0	26	3	47.1	−5.18	5.48	4.32	No
**Afacifenacin**	481.52	5	1	6	5	10	8	0	30	1	44.81	−6.63	5.91	4.58	No*
**Piketoprofen**	344.41	4	1	5	3	6	4	0	21	1	62.29	−4.58	3.77	3.69	Yes
**Bemcentinib**	506.66	8	3	3	7	15	8	0	41	1	101.74	−3.45	4.73	3.92	No*
**Fluazuron**	506.21	6	2	8	3	6	13	0	20	0	80.32	−7.63	5.26	4.16	No
**Heliomycin**	376.36	6	4	0	5	16	6	0	25	0	115.06	−5.55	4.71	1.86	Yes
**Etoposide**	588.56	13	3	5	7	16	13	0	37	10	160.83	−3.57	1.69	1.79	No*
**Optimal range**	**100–600**	**0–12**	**0–7**	**0–11**	**0–6**	**0–18**	**1–15**	**−4 − 4**	**0–30**	**≤ 2**	**0–140**	**-4-0.5**	**0–3**	**1–3**	**Yes**

Listed are the physicochemical properties of the ligands with highest binding affinity for each of the eleven candidate target proteins

*nHA* Number of hydrogen bond acceptors, *nHD* Number of hydrogen bond donors, *nRot* Number of rotatable bonds, *nRing* Number of rings, *MaxRing* Number of atoms in the biggest ring, *nHet* Number of heteroatoms, *fChar* Formal charge, *nRig* Number of rigid bonds, *TPSA* Topological polar surface area, *logS* Log of the aqueous solubility, *logP* Log of the octanol/water partition coefficient, *logD* LogP at physiological pH of 7.4, *RO5* Rule of five

The rule of five scores and integrates values for molecular weight, nHA, nHD, and logP for a synopsis of orally active drug-likeness. Asterisks mark ligands that do not fulfill the RO5 but have been applied orally in clinical studies. Molecular weight and RO5 were retrieved from ChEMBL, the other properties were predicted by ADMETlab

Eight of the eleven putative targets in acanthocephalans were likely to have enzymatic activity according to ECPred, and ten of the target molecules contained PFAM motifs (Table 1, Supplementary Tables S3, S4). Furthermore, three of the ten compounds were labelled according to the Globally Harmonized System (GHS). These were etoposide (irritant, health hazard), tadalafil (irritant), and fluazuron (environmental hazard) (Supplementary Table S6). Still, these limitations do not necessarily preclude their repurposing in acanthocephaliasis (see Discussion). Indeed, the usability of all ten agents as drugs was reflected in the fact that most of the parameters giving absorption, distribution, metabolism, excretion, and toxicity (ADMET) were in the optimal range (Table 2). Furthermore, eight of the agents fulfilled the rule of five (RO5), indicating suitability for oral administration, or have already been successfully administered per os in clinical trials (see asterisks in RO5 column of Table 2). This requirement can be considered almost mandatory for the treatment of fish in aquaculture. In sum, a total of five compounds are likely to be most promising for future in vitro testing in acanthocephaliasis. These are pranazepide, piketoprofen, and heliomycin, in addition to above-mentioned derquantel and tadalafil.

## Discussion

Present results demonstrate the utility of a workflow for identifying putative ligands to target proteins in parasites. The approach is based on genomic, transcriptomic, and proteomic data, followed by 3D structure and binding site prediction, virtual ligand screening, and ADMET property prediction. Applied to fish-parasitizing acanthocephalans, we identified eleven proteins the blocking of which should enable an effective and specific control of these parasites. Five of the ten identified ligands appear to be particularly promising for further testing in vitro and in fish aquacultures.

### Candidate target identification in parasites

The filtering workflow was designed to converge on druggable targets in fish-parasitizing acanthocephalans (Fig. 1; Supplementary Fig. S1). One requirement was elevated and little varying up to constant transcript abundance in alternative host species providing different physiological environments to the parasite. This suggests targetability in all acanthocephalan specimens. Confirmation of high abundance on the protein level and, especially, in the acanthocephalan body wall should increase their accessibility to orally administered compounds. We expect this to be a requirement for an effective control of gutless acanthocephalans, which take up nutrients and minerals via the surface of the body wall [46–48]. A corresponding example is the drug loperamid, which has been shown to enter acanthocephalans via the surface of the tegument making up the outermost part of the body wall upon oral administration to infected pigs [58]. In addition, combating acanthocephalans, so to speak, at the point of entry should be more likely to succeed than targeting proteins in inner organs. In addition, we consider it beneficial for the specificity of acanthocephalan control if target proteins lack homologues in non-acanthocephalan taxa. However, this criterion is not absolute, and we here allowed for the retention of parasite proteins showing high sequence divergence compared to putative homologues in other taxa. Such homology should even be advantageous since corresponding targets could be involved in basic pathways the blocking of which should strongly affect the parasites. On the contrary, we regarded the presence of homologues in two *Neoechinorhynchus* species as mandatory. This criterion ensured the determination of target proteins that are conserved among fish-parasitizing palaeacanthocephalans and eoacanthocephalans from distant geographic regions (South America and Eurasia). More importantly, one of the species included was *N. buttnerae*, which is the economically most important acanthocephalan pest species in fish farms. In fact, this species is the major problem in South American limnocultures of fish, decreasing the yield of aquaculture farms by up to 90% [8, 45, 59–64]. Thus, confirmation of the candidate targets in *N. buttnerae* has direct implications for the practical use of the present results.

### Drugs against acanthocephalans

We consider present protein structure models as reasonably reliable for the prediction of binding sites and subsequent ligand screening. This is because the 3D models were obtained using the two most accurate de novo modeling tools currently available, Alphafold2 and RoseTTAFold [31, 73, 79]. Among the ten ligands which should bind strongest to our eleven targets in acanthocephalans (Table 1, Table 2), a notable hit was derquantel, which would be a candidate for drug repurposing. The compound is an antagonist of N-acetylcholine receptors in nematodes [80, 81] and as such is contained in a commercial dewormer for sheep, marketed under the label Startect (Zoetis Australia Pty Ltd.; Licence: VPA10387/066/001.2017). Oral administration of Startect was shown to be highly effective (up to 100%) against diverse nematode parasites of the gastrointestinal tract and respiratory system [80, 82]. In virtual ligand screening, derquantel bound most strongly to one of the prime target candidates in acanthocephalans and second most strongly to another one (Table 1). Thus, application of Startect against fish-parasitizing acanthocephalans seems feasible. On the other hand, the second active ingredient of Startect, abamectin, has insecticide and acaricide activity [83, 84]. Thus, dissipation of Startect into the environment might be problematic and the mere administration of derquantel might be the better choice in fish.

Another compound for which high affinity was predicted to two of our eleven candidate targets was tadalafil, a phosphodiesterase (PDE) inhibitor (Table 2). Strikingly, inhibitors of PDE were previously suggested to have potential to control parasitic nematodes due to their disruptive effects on the *Caenorhabditis elegans* life cycle and nematode-specific active binding sites [85, 86]. In humans, tadalafil competes with the secondary messenger cGMP for binding sites in phosphodiesterase 5 (PDE5), thus relaxing the smooth musculature in several organs [87]. Such a mechanism should adversely affect acanthocephalans, in which the entire musculature is of the smooth type [88]. Also, tadalafil could interfere with acanthocephalan energy metabolism as suggested by PFAM motifs for phosphatidylglycerophosphatase (PGP) activity in both predicted target proteins (Supplementary Table S4). If true, this would be a novel mechanism for tadalafil. Not least, approved use in humans illustrates that the irritant potential of tadalafil according to the GHS is quite manageable.

Anthelmintic potential seems possible for piketoprofen, heliomycin, and pranazepide too, due to their anti-inflammatory, RNA synthesis antagonist, and cholecystokinin receptor type A antagonist activity, respectively [89–91]. For two of the remaining five compounds in Table 2, there is evidence for effectiveness against helminths once more. Thus, fluazuron is used in conjunction with above-mentioned abamectin, in the control of the gastrointestinal nematodes infecting cattle [92]. Furthermore, etoposide can induce cell cycle arrest at the G2/M phase and apoptotic cell death in *C. elegans* [93]. Also, widely constant transcript abundances suggest that the predicted acanthocephalan target proteins should be readily addressable by fluazuron and etoposide (Supplementary Table S1). Yet, both compounds, fluazuron and etoposide, might not be the first choice for acanthocephalan control, given their GHS labels (see Results). Still, all the ten agents in Table 2 result from conservative filtering of their potential target molecules. They all should have potential for use as effective agents against in acanthocephalans. Their application should additionally enable a more specific killing of acanthocephalans than would be achieved with niclosamide or benzimidazole derivates [9, 12, 13].

## Conclusions

The development of drugs for parasite control usually takes many years and can easily cause enormous costs for pre-clinical and clinical trials, environmental impact assessment, approval, and the establishment of industrial production. Here, we present a bioinformatics workflow intended to reduce time and cost that is also applicable to non-model parasites for which little functional information is available. The entire workflow includes the identification of candidate targets in parasites and subsequent virtual screening for ligands. Detailed steps are quantitative transcriptome and proteome analyses, prediction of 3D protein structures and binding sites, and virtual database screening for binding compounds. In addition to the novel combination of individual analyses, the approach to the best of our knowledge utilizes for the first time in target identification environmental variation which the parasite is exposed to in definitive and accidental hosts.

Application of the workflow to fish-parasitizing acanthocephalans led to the identification of eleven top-ranked candidate target proteins (Table 1). Compounds predicted to bind to them already exist (Table 2), whereby five appear to be particularly promising according to ADMET, GHS and RO5 classifications: derquantel, tadalafil, pranazepide, piketoprofen, and heliomycin. We take it as confirmation of the usefulness of the present workflow that one of these compounds, derquantel, is an established nematocidal anthelmintic [80, 82]. A second compound, tadalafil, inhibits PDE5 and thus interferes with a metabolic pathway previously suggested to be a promising target for novel nematocidal anthelmintics [85]. Nematocidal effectiveness of two additional compounds, namely fluazuron and etoposide, further corroborates the usefulness of the workflow [92, 93].

Future simulations might shed light on the detailed nature of interaction between the candidate targets and ligands mentioned above. Probably, it will be revealing to examine the extent to which the 3D models determined here represent the active protein structure. Moreover, enabling fit induction by the ligand might uncover hitherto hidden binding sites in acanthocephalan proteins [94, 95]. Such analyses might lead to additional candidate targets but, to our estimation, will unlikely change that the current ones are worthwhile further evaluation, which will also have to include the testing of tolerability, ecotoxicology, specificity, and effectiveness in vitro and in vivo. The candidate targets listed in Table 1 may additionally be used as starting points for screening databases of compounds for which less knowledge is available. Obviously, validation of such compounds would raise costs and take time. But the present filtering of target candidates should increase the probability that any agents enable an effective control of acanthocephalans, whether the drugs will be repurposed or newly developed. Relative to broad-spectrum anthelminthics such as niclosamide and benzimidazole derivatives [9, 12, 13], any novel anti-parasitic strategy developed on the candidate targets in Table 1 should also allow for a specific control of acanthocephalans in fish aquacultures. These efforts might lead to new strategies against acanthocephaliasis, the main current obstacle in establishing successful fish aquaculture in South America (e.g., [61]). Although demonstrated here in acanthocephalans, this novel application can be transferred to a broad range of parasitic taxa [96]. For this purpose, the target determination workflow can be accessed via the Galaxy web server (Supplementary Fig. S1).

## Methods

### Sampling and sequencing

Fish were caught and sacrificed by authorized persons immediately prior to excision of acanthocephalans from the guts. As for the processing of the eoacanthocephalans *N. agilis* and *N. buttnerae* we refer to Supplementary Notes S1 and S2. Central to present transcriptome analyses was the palaeacanthocephalan *P. laevis s.l.* [97]. We analyzed *N* = 20 worms, with 10 specimens (5 males, 5 females) from common barbel and 10 specimens (5 males, 5 females) from European eel. All samples were sequenced as 75 bp single-end reads on an Illumina HiSeq 2500. Raw sequences are available at the EMBL Nucleotide Sequence Database (ENA) repository under accession numbers ERS7302868–87 in project PRJEB47442. Adapter sequences and low-quality parts of the sequences were trimmed with Trimmomatic v0.39 [98]. For more details of sampling and sequencing see [99]. While we aimed to filter out proteins with constant abundance across conditions in the present study, the referenced study demonstrates overall differing proteomic profiles in male and female worms from different hosts. We take this as a confirmation that sample processing did not compromise quantitative analyses of transcriptome data.

### Differential gene expression analysis

As reference we used the *P. laevis* transcriptome published recently under NCBI GenBank accession number GIBA00000000.1 [65]. This transcriptome shotgun assembly was generated by Trinity v2.4.0 [100] from male, female and juvenile specimens. Transcriptome contigs were checked for bacterial contamination by blastn searches in BLAST+ v2.10.0 [101] against 21,820 bacterial reference genomes downloaded from NCBI. All contigs with hits below the E-value cutoff of 1e-20 were removed from the assembly for subsequent analyses. To check for congruence of our RNA-Seq datasets with the reference transcriptome we mapped all datasets with BBMap v38.73 (https://sourceforge.net/projects/bbmap/) to the reference. Since 92–96% of reads mapped successfully under default settings, the transcriptome seems to be quite complete and serves as a useful resource for the analysis.

Transcript quantification was done with the RSEM v1.3.3 [102] software package and the reference transcriptome described above. We applied Bowtie 2 v2.4.1 [103] mapping with settings optimized and implemented for RSEM downstream analysis. The rsem-calculate-expression script was applied with -calc-ci option for calculation of confidence intervals during calculation of relative transcript abundances.

Transcript abundance analyses were carried out with the Bioconductor package DESeq2 v1.28.1 [104] in R v4.0.3 (https://www.gbif.org/tool/81287/r-a-language-and-environment-for-statistical-computing) [105]. Since we were interested in approximating gene expression rather than inferring abundances of single transcripts, we summed up read counts across transcript variants (based on Trinity annotation). Corresponding integers were used as input for DESeq2 analyses between four pairs of comparison: (1) female worms from barbel vs. male worms from barbel, (2) female worms from eel vs. male worms from eel, (3) male worms from barbel vs. male worms from eel, and (4) female worms from barbel vs. female worms from eel. We applied the DESeq2 alternative hypothesis ‘lessAbs’, which tests for genes having transcript read counts within user-defined boundaries.

### Identification of candidate targets

As an approximation of targetability, we only kept genes having at least 50 transcript reads in each of the 20 samples. Then, we extracted all significantly unregulated genes, according to likewise transcript read counts in all four DESeq2 pairs of comparisons delineated above. In detail, retention of a gene required a maximum log fold change of 1.50 between at least two of the four groups of comparison (adjusted *p*-value ≤0.05, each). Corresponding genes were regarded to be expressed independently of sex and host, suggestive of their constitutive expression.

Open reading frames (ORFs) were extracted from candidate target transcripts running getorf within EMBOSS v6.5.7 [106] with default settings. Only ORFs with translated sequences of at least 30 amino acids were kept for subsequent filtering. The resulting protein sequences were further investigated. We especially searched for orthologues of candidate target proteins in newly assembled draft genomes of two additional acanthocephalan species (*N. buttnerae*, *N. agilis*) (see Supplementary Notes S1 & S2; Supplementary Tables S7 & S8). Only sequences yielding tblastn hits below an E-value cutoff of 1e-50 were regarded to be conserved within fish-parasitizing acanthocephalans, and hence were kept.

Exclusion of sequences with similar sequences in non-acanthocephalan taxa was accomplished by blastp searches against the SwissProt database. For the same reason, we carried out tblastn searches against the genomes of six species from Bdelloidea, Monogononta, and Seisonidea, i.e., the three other higher-ranked taxa within the Rotifera-Acanthocephala clade commonly referred to as Syndermata or just Rotifera (*sensu* *lato*): *Adineta vaga* (GCA_000513175.1), *Adineta ricciae* (GCA_900240375.1), *Brachionus calyciflorus* (GCA_002922825.1), *Brachionus koreanus* (GCA_009177125.1), *Brachionus plicatilis* (GCA_010279815.1), and *Seison nebaliae* (PRJEB43415). All sequences yielding hits with E-values ≤1e-50 were regarded evolutionarily conserved between Acanthocephala and the taxa compared. Corresponding hits were excluded from further analyses.

### Protein quantification by mass spectrometry

Protein isolation used the body walls of 192 worms (Supplementary Table S9) freed from the proboscis and emptied from internal organs and body fluid by gentle pressure. Five pools of body walls were boiled in lithium dodecyl sulfate buffer (Life Technologies, Carlsbad, CA, USA). Proteins were separated by polyacrylamide gel electrophoresis on a Novex NuPAGE 4–10% gel (Thermo Fischer Scientific, Waltham, MA, USA). Upon mincing of gel pieces, disulfide bonds were reduced with 10 mM DTT (Sigma-Aldrich, St Louis, MO, USA) at 55 °C, followed by alkylation with 55 mM iodoacetamide (Sigma-Aldrich). In-gel digestion was done with mass spectrometry-grade trypsin (Sigma-Aldrich) at 37 °C overnight. Peptides were eluted from the gel with acetonitrile, which was removed in a concentrator (Eppendorf SE, Hamburg, Germany) prior to loading on an Empore C18 StageTip (3 M Purification Inc., St Paul, MN, USA).

The measurement was performed on an EASY-nLC 1200 HPLC coupled online to an Orbitrap Exploris 480 mass spectrometer (Thermo Fischer Scientific), operated in data-dependent acquisition mode with a top20 method. During the 120 min measurement, peptides were eluted with an optimized 5–40% acetonitrile/water gradient. Raw files were processed with MaxQuant v1.6.5.0 [107, 108] using the settings: digestion = trypsin specific, max missed cleavages = 2, peptide FDR = 0.01, protein FDR = 0.01, variable modifications = oxidation (M) and acetylation (protein N-terminus), fixed modification = carbamidomethylation (C), match between runs = activated, iBAQ quantitation = activated. The search to homologize protein to mRNA sequences was performed against all ORF sequences derived from the transcriptome assembly of *P. laevis*.

### Correlation analyses

Abundances of matched proteins (iBAQ) and transcripts (transcript read counts) was carried out with Excel 2019 (Microsoft).

### Protein properties and structure modeling

Sequences of candidate target proteins were screened for PFAM protein motifs and domain features [109] at Kyoto University Bioinformatics Center’s GenomeNet MOTIF search (https://www.genome.jp/tools/motif/; accessed 2021-09-01) and for PROSITE protein domains and functional sites [110] at the Swiss Institute of Bioinformatics’ Resource Portal (https://prosite.expasy.org/; accessed 2021-09-15). Additionally, potential enzyme functions were predicted by ECPred [111].

Protein’s grand average of hydropathy [112] was calculated by the Sequence Manipulation Suite’s GRAVY algorithm implementation (https://www.bioinformatics.org/sms2/protein_gravy.html). N-terminal pre-sequences were predicted by TargetP v2.0 [113] and transmembrane topologies by DeepTMHMM (https://biolib.com/DTU/DeepTMHMM). Prediction of subcellular localization based on sequence information was accomplished by the deep learning algorithm DeepLoc v1.0 [114].

Protein 3D structures were modeled with AlphaFold2 [31] as executed by a Jupyter Notebook [115] on Google Colab (https://colab.research.google.com/github/sokrypton/ColabFold/blob/main/AlphaFold2.ipynb) [116]. We applied default settings, including MMseqs2 [117] for sequence alignment. The best out of five calculated models was used for further analysis, based on the ranking by the average predicted Local Distance Difference Test value (pLDDT; see Suppl. Methods of AlphaFold2). For validation, protein 3D structures were additionally modeled with RoseTTAFold [79] applying default settings. Results from both modelers were compared using the University of Helsinki’s Dali protein structure comparison server [74, 118].

### Binding site prediction, ligand screening, and docking

Protein-ligand binding site prediction was carried out with COACH-D [76] on protein PDB files as generated by AlphaFold2. Each protein’s best binding site was subjected to ligand screening, plus all secondary sites with confidence scores (C-score) up to 0.3 lower than the site ranked first. High-throughput virtual ligand screening was performed using AutoDock Vina v1.2.0 [77], implemented in MTiOpenScreen [78]. Settings were: Demonstration mode = No, Protein Receptor = PDB, Is lead-like = Yes, Grid calculation = Custom parameters. AlphaFold2-derived PDB files and COACH-D coordinates of the binding site were used as input. We screened against the Drugs-lib compound database that contains 21,276 drugs that are either approved or have been used in clinical trials [78].

Ligands predicted to bind with most favorable free energy to binding sites in the candidate target proteins were further evaluated in the online databases ChEMBL [119], ClinicalTrials.gov (https://clinicaltrials.gov/), DrugBank [120], and PubChem [121]. Special emphasis was given to published indications, known/predicted molecular targets, mode of administration, resp. the fulfilment of the RO5, and annotations within the GHS classification system (Supplementary Table S6). Additionally, physicochemical absorption, distribution, metabolism, excretion, and toxicity (ADMET) properties were predicted using ADMETlab 2.0 [122].

## Supplementary Information

**Additional file 1: Supplementary Note S1.** Assembly of *Neoechinorhynchus buttnerae* draft genome. **Supplementary Note S2.** Assembly of *Neoechinorhynchus agilis* draft genome. **Supplementary Table S1.** Transcript abundance differences of candidates. **Supplementary Table S2.** Amino acid composition of candidate target proteins. **Supplementary Table S3.** Properties of target proteins. **Supplementary Table S4.** PFAM motifs of target proteins. **Supplementary Table S5.** 3D structure prediction of target proteins. **Supplementary Table S6.** Virtual ligand screening results. **Supplementary Table S7.** Bacterial genome assemblies used for data decontamination. **Supplementary Table S8.** Annotation of the mitochondrial genome of *N. buttnerae*. **Supplementary Table S9.** Specimens used for mass spectrometry. **Supplementary Table S10.** Standard InChI keys and 2D structures of candidate ligands. **Supplementary Figure S1.** Target identification workflow at Galaxy.

**Additional file 2: Supplementary Tables S3-S6.**

## Data Availability

The transcriptome datasets are available in the EMBL Nucleotide Sequence Database (ENA) repository under the accession number PRJEB47442 (ERS7302868–87). The presented workflow is accessible via the Galaxy server under the URL tinyurl.com/yx72rda7. Supplementary Fig. S1 provides a corresponding overview.
