# Supplementary material for: Identification of antiparasitic drug targets using a multi-omics workflow in the acanthocephalan model
Source: BMC Genomics. 2022 Sep 30;23:677. doi: 10.1186/s12864-022-08882-1 (PMC9523657; doi:10.1186/s12864-022-08882-1)
Supplement: Supplementary file 1 — Additional file 1: Supplementary Note S1. Assembly of Neoechinorhynchus buttnerae draft genome. Supplementary Note S2. Assembly of Neoechinorhynchus agilis draft genome. Supplementary Table S1. Transcript abundance differences of candidates. Supplementary Table S2. Amino acid composition of candidate target proteins. Supplementary Table S3. Properties of target proteins. Supplementary Table S4. PFAM motifs of target proteins. Supplementary Table S5. 3D structure prediction of target proteins. Supplementary Table S6. Virtual ligand screening results. Supplementary Table S7. Bacterial genome assemblies used for data decontamination. Supplementary Table S8. Annotation of the mitochondrial genome of N. buttnerae. Supplementary Table S9. Specimens used for mass spectrometry. Supplementary Table S10. Standard InChI keys and 2D structures of candidate ligands. Supplementary Figure S1. Target identification workflow at Galaxy. [file 12864_2022_8882_MOESM1_ESM.docx]

Supplementary Information to

Identification of antiparasitic drug targets using a multi-omics workflow in the acanthocephalan model

Hanno Schmidt, Katharina Mauer, Manuel Glaser, Bahram Sayyaf Dezfuli, Sören Lukas Hellmann, Ana Lúcia Silva Gomes, Falk Butter, Rebecca C. Wade, Thomas Hankeln, Holger Herlyn

Supplementary Notes

[Supplementary Note S1 – Assembly of *Neoechinorhynchus buttnerae* draft genome 2](#_Toc109307730)

[Supplementary Note S2 – Assembly of *Neoechinorhynchus agilis* draft genome 3](#_Toc109307731)

Supplementary Tables

[Supplementary Table S1 – Transcript abundance differences of candidates 5](#_Toc103683468)

[Supplementary Table S2 – Amino acid composition of candidate target proteins 6](#_Toc103683469)

[Supplementary Table S3 – Properties of target proteins 7](#_Toc103683470)

[Supplementary Table S4 – PFAM motifs of target proteins 7](#_Toc103683471)

[Supplementary Table S5 – 3D structure prediction of target proteins 7](#_Toc103683472)

[Supplementary Table S6 – Virtual ligand screening results 7](#_Toc103683473)

[Supplementary Table S7 – Bacterial genome assemblies used for data decontamination 7](#_Toc103683474)

[Supplementary Table S8 – Annotation of the mitochondrial genome of N. buttnerae 9](#_Toc103683475)

[Supplementary Table S9 – Specimens used for mass spectrometry 10](#_Toc103683476)

[Supplementary Table S10 – Standard InChI keys and 2D structures of candidate ligands 11](#_Toc103683477)

Supplementary Figures

[Supplementary Figure S1 – Target identification workflow at Galaxy 13](#_Toc109895091)

References………………………………………………………………………………………………………………………………………. 14

Supplementary Notes

# Supplementary Note S1 – Assembly of *Neoechinorhynchus buttnerae* draft genome

*Neoechinorhynchus buttnerae* worms were sampled in 2018 from tambaqui (*Colossoma macropomum*) held in limnocultures close to Manaus, Brazil. Genomic DNA of 35 specimens was isolated by innuPREP DNA/RNA Mini Kit (Analytik Jena, Jena, Germany) according to the manufacturer’s protocol. DNA was custom-sequenced as 150 bp Illumina paired-end reads with an insert size of 350 bp. Sequencing resulted in 306 million read pairs. Sequence reads were quality processed using Trimmomatic v0.39 [1] with ILLUMINACLIP:2:30:10, LEADING:3, TRAILING:3, SLIDINGWINDOW:4:15, MINLEN:40 in paired end mode. Raw and quality processed reads were quality checked with FastQC v0.11.9 [2]. Reads were purged from mitochondrial sequences by k-mer filtering with BBDuck from the BBTools program suite v38.73 [3] using the complete *N. buttnerae* mitochondrial genome as reference (assembled in the present study; see below). To minimize contamination risk, reads were mapped with BBMap from BBTools to 31 bacterial genomes (Supplementary Table S7) representing the most common bacterial clades/species in fish gut microbiomes according to a recent review article [4]. On average 0.02% of the reads mapped. Only intact read pairs that did not map to the bacterial genomes were used for assembly with Megahit v1.2.9 [5] with custom settings (k-min=25, k-max=149, k-step=10, min-count=4, prune-level=3, no-mercy). Sequences < 1000 bp were removed from the resulting assembly. The nuclear genome assembly consisted of 15,849 contigs with a total length of 50,233,622 bp, an N50 value of 4,538 bp, and a mean length of 3,169 bp. The longest sequence was 322,622 bp. Backmapping of all reads represented in the assembly was done with BBMap. This resulted in 98.4% mapped reads, illustrating a very high completeness of the genome sequence despite its draft status. Completeness of the gene content was assessed with BUSCO v5.2.1 [6], using metazoan core orthologs and ‘--long’ enabled. *Schistosoma mansoni* was selected as Augustus [7] gene model species. In doing so, 55.6% of the core orthologs were found in the genome, which is similar to the 61% reported for the *P. laevis* genome [8].

For assembling the mitochondrial genome of *N. buttnerae*, we ran MITObim v1.9.1 [9] with the mitochondrial genome sequence of *Paratenuisentis ambiguus* as baiting reference, Geneious v2019.2 (Biomatters Limited, New Zealand) with 4% of the total reads randomly extracted, and CLC Genomics Workbench v12.0 (Qiagen, Netherlands) with 10% of the total reads randomly extracted. Upon alignment generation with MAFFT [10], a consensus sequence was derived with BioEdit v7.0.5.3 [11]. Annotation of the consensus sequence used MITOS [12] with genetic code ‘invertebrates’, MitoZ [13] with ‘genetic_code 5’ and ‘--clade Arthropoda’, and MFannot [14] with genetic code ‘invertebrates’ once more. Gene boundary determination was validated in NCBI ORF-Finder results with ‘5 – invertebrate mitochondrial’ and ‘ATG and alternative start codons’. Annotation of tRNA genes was carried out with the programs ARWEN [15] with ‘metazoan mitochondrial tRNAs’, and DOGMA [16] with ‘invertebrates’ and ‘COVE threshold = 0’. The resulting mitochondrial genome sequence had 13,255 bp and the common metazoan repertoire of 36 genes [17] could reliably be annotated (Supplementary Table S8). Gene order is the same as for *P. laevis* except for *trnH* which localized between a *nad1*-adjacent non-coding region and rrnS instead of residing between *nad4* and *nad5*.

# Supplementary Note S2 – Assembly of *Neoechinorhynchus agilis* draft genome

*Neoechinorhynchus agilis* worms were sampled in 2020 from 15 thinlip mullets (*Chelon ramada*) caught in an Adriatic lagoon close to Ferrara, Italy. Genomic DNA of 50 specimens was isolated by DNeasy Blood & Tissue Kit (Qiagen, Hilden, Germany) according to the manufacturer's protocol. DNA was sequenced by StarSEQ (Mainz, Germany) on an Illumina NextSEQ 500 as 150 bp paired-end reads with a major fragment size of 200-800 bp. Sequencing resulted in 52 million read pairs. Sequence reads were quality processed using Trimmomatic with ILLUMINACLIP:2:30:10, LEADING:3, TRAILING:3, SLIDINGWINDOW:4:15, MINLEN:40 in paired end mode. Raw and quality processed reads were quality checked with FastQC. Reads were then mapped with BBMap against the same 31 bacterial genomes as in *N. buttnerae* (Supplementary Table S7). On average 0.002% of the reads mapped. Only intact read pairs that did not map to the bacterial genomes were used for assembly with Megahit with the same settings as for *N. buttnerae* (see Supplementary Note S1). Sequences < 1000 bp were removed from the assembly. The nuclear genome assembly consisted of 6,930 contigs with a total length of 37,481,652 bp, an N50 value of 10,384 bp, and a mean length of 5,409 bp. The longest sequence had 96,976 bp. Backmapping of all reads used for assembly with BBMap resulted in 99.4% mapped reads, illustrating a very high completeness of the genome sequence despite its draft status. Completeness of the gene content was assessed with BUSCO as detailed for *N. buttnerae* (see Supplementary Note S1). In the draft genome, 55.7% of the metazoan core orthologs were found. As mentioned above, the draft genome of *P. laevis* was found before to contain a likewise low percentage of metazoan core orthologs (61%), which thus seems to be a general characteristic of highly derived acanthocephalans [8].

Supplementary Tables

Supplementary Table S1 – Transcript abundance differences of candidate targets

Shown are the log2 fold changes for the final eleven candidate targets (padj < 0.05) in the four pairs of comparison analyzed using DESeq2’s alternative hypothesis testing (lessAbs).

| **Candidate target ID** | **Male vs. female from barbel** | **Male vs. female from eel** | **Females from eel vs. barbel** | **Males from eel vs. barbel** |
| --- | --- | --- | --- | --- |
| 1609 | 1.75 | 1.25 | 2.50 | 1.25 |
| 4617 | 3.50 | 1.00 | 3.00 | 1.50 |
| 5995 | 1.50 | 1.00 | 3.25 | 2.50 |
| 7137 | 1.00 | 0.75 | 0.75 | 0.75 |
| 8627 | 1.50 | 0.75 | 2.00 | 1.00 |
| 8750 | 1.25 | 0.75 | 0.75 | 0.75 |
| 8763 | 1.25 | 0.75 | 1.50 | 0.75 |
| 9169 | 0.75 | 0.75 | 1.25 | 1.25 |
| 9190 | 1.00 | 0.75 | 1.00 | 0.75 |
| 9257 | 0.75 | 0.75 | 0.75 | 0.75 |
| 9684 | 1.25 | 0.75 | 2.75 | 2.25 |

Supplementary Table S2 – Amino acid composition of candidate target proteins

Amino acid composition was calculated for 52 intermediate candidate target proteins (actual %) and compared to average values (typical %) of 614 eukaryotic proteomes [18]. Positive correlation between compositions was strong (0.88) and highly significant (p = 3.6e-07; Student's *t*-test).

| **Amino acid** | **Actual %** | **Typical %** | **Difference** |
| --- | --- | --- | --- |
| Alanine | 5.32 | 7.63 | -2.31 |
| Cysteine | 1.90 | 1.76 | 0.14 |
| Aspartic acid | 6.10 | 5.40 | 0.70 |
| Glutamic acid | 5.68 | 6.42 | -0.74 |
| Phenylalanine | 4.19 | 3.87 | 0.32 |
| Glycine | 5.25 | 6.33 | -1.08 |
| Histidine | 2.74 | 2.44 | 0.30 |
| Isoleucine | 7.26 | 5.10 | 2.16 |
| Lysine | 6.46 | 5.64 | 0.82 |
| Leucine | 8.38 | 9.29 | -0.91 |
| Methionine | 2.15 | 2.25 | -0.10 |
| Asparagine | 6.11 | 4.28 | 1.83 |
| Proline | 4.50 | 5.41 | -0.91 |
| Glutamine | 4.36 | 4.21 | 0.15 |
| Arginine | 5.11 | 5.71 | -0.60 |
| Serine | 9.26 | 8.34 | 0.92 |
| Threonine | 5.10 | 5.56 | -0.46 |
| Valine | 5.58 | 6.20 | -0.62 |
| Tryptophan | 0.72 | 1.24 | -0.52 |
| Tyrosine | 3.83 | 2.87 | 0.96 |

Supplementary Table S3 – Properties of target proteins

Supplementary Table S1 is available in the separate Excel spreadsheet file Additional Information 2.

Supplementary Table S4 – PFAM motifs of target proteins

Supplementary Table S2 is available in the separate Excel spreadsheet file Additional Information 2.

Supplementary Table S5 – 3D structure prediction of target proteins

Supplementary Table S3 is available in the separate Excel spreadsheet file Additional Information 2.

Supplementary Table S6 – Virtual ligand screening results

Supplementary Table S4 is available in the separate Excel spreadsheet file Additional Information 2.

Supplementary Table S7 – Bacterial genome assemblies used for data decontamination

Reference genomes of 31 bacterial species used for read filtering. The corresponding species were previously found to be abundant in fish gut microbiomes [4].

| **Bacterial species** | **Accession number** | **Bacterial species** | **Accession number** |
| --- | --- | --- | --- |
| *Cetobacterium ceti* | GCA_900167275.1 | *Pseudomonas aeruginosa* | GCA_000006765.1 |
| *Cetobacterium somerae* | GCA_902375135.1 | *Bacillus subtilis* | GCA_000009045.1 |
| *Fusobacterium necrophorum* | GCA_003019715.1 | *Bacillus clausii* | GCA_000009825.1 |
| *Fusobacterium nucleatum* | GCA_013137915.1 | *Staphylococcus aureus* | GCA_000013425.1 |
| *Escherichia coli* | GCA_000005845.2 | *Clostridium perfringens* | GCA_000013285.1 |
| *Shewanella putrefaciens* | GCA_000016585.1 | *Desulfovibrio vulgaris* | GCA_000166115.1 |
| *Corynebacterium matruchotii* | GCA_000175375.1 | *Enterovibrio calviensis* | GCA_000621165.1 |
| *Agathobaculum desmolans* | GCA_000701665.1 | *Delftia acidovorans* | GCA_000741825.1 |
| *Vibrio harveyi* | GCA_000770115.2 | *Alteromonas macleodii* | GCA_000808635.1 |
| *Bacteroides fragilis* | GCA_001286525.1 | *Lacticaseibacillus rhamnosus* | GCA_002848015.1 |
| *Photobacterium phosphoreum* | GCA_002954725.1 | *Acinetobacter junii* | GCA_002761875.1 |
| *Faecalibacterium prausnitzii* | GCA_003312465.1 | *Aeromonas salmonicida* | GCA_012931585.1 |
| *Flavobacterium psychrophilum* | GCA_003433335.1 | *Lactococcus lactis* | GCA_016406265.1 |
| *Mycoplasma putrefaciens* | GCA_900476175.1 | *Achromobacter xylosoxidans* | GCA_013343135.1 |
| *Propionibacterium freudenreichii* | GCA_900087655.1 | *Psychrobacter maritimus* | GCA_904846345.1 |
| *Papillibacter cinnamivorans* | GCA_900176335.1 |  |  |

Supplementary Table S8 – Annotation of the mitochondrial genome of N. buttnerae

The annotation was generated by MITOS and manually curated with additional input (see Supplementary Note S1). Amino acids in tRNA gene names are given in single letter code and with respective anticodon in parentheses. Cox1-3: cytochrome c oxidase subunits 1-3; nad1-6: NADH dehydrogenase subunits 1-6; atp6: ATP synthase subunit 6; cob: cytochrome b; rrnS: 12S rRNA; rrnL: 16S rRNA; NCR: non-coding region.

| **Gene name** | **Start [bp]** | **Stop [bp]** | **Length [bp]** |
| --- | --- | --- | --- |
| cox1 | 1 | 1,531 | 1531 |
| trnG (UCC) | 1,532 | 1583 | 52 |
| trnQ (UUG) | 1,584 | 1,626 | 43 |
| trnY (GUA) | 249 | 1154 | 906 |
| rrnL | 1,673 | 2,540 | 868 |
| trnL1 (UAG) | 2,541 | 2,591 | 51 |
| nad6 | 2,592 | 3,020 | 429 |
| trnD (GUC) | 3,019 | 3,069 | 51 |
| atp6 | 3,159 | 3,719 | 561 |
| nad3 | 3,720 | 4,043 | 324 |
| trnW (UCA) | 4,045 | 4,103 | 59 |
| NCR1 | 4,104 | 4,436 | 333 |
| trnV (UAC) | 4,437 | 4,492 | 56 |
| trnK (UUU) | 4,475 | 4,524 | 50 |
| trnE (UUC) | 4,514 | 4,564 | 51 |
| trnT (UGU) | 4,554 | 4,611 | 58 |
| trnS2 (UGA) | 4,600 | 4,648 | 49 |
| nad4l | 4,649 | 4,891 | 243 |
| nad4 | 4,891 | 6,144 | 1,254 |
| nad5 | 6,146 | 7,762 | 1,617 |
| trnL2 (UAA) | 7,762 | 7,814 | 53 |
| trnP (UGG) | 7,815 | 7,882 | 68 |
| cob | 7,872 | 8,961 | 1,090 |
| nad1 | 8,965 | 9,837 | 873 |
| trnI (GAU) | 9,837 | 9,880 | 44 |
| NCR2 | 9,881 | 10,070 | 190 |
| trnH (GUG) | 10,071 | 10,124 | 54 |
| trnM (CAU) | 10,161 | 10,221 | 61 |
| rrnS | 10,222 | 10,806 | 585 |
| trnF (GAA) | 10,807 | 10,871 | 65 |
| cox2 | 10,900 | 11,472 | 573 |
| trnC (GCA) | 11,471 | 11,521 | 52 |
| cox3 | 11,540 | 12,257 | 718 |
| trnA (UGC) | 12,258 | 12,310 | 53 |
| trnR (UCG) | 12,311 | 12,369 | 59 |
| trnN (GUU) | 12,359 | 12,412 | 54 |
| trnS1 (ACU) | 12,409 | 12,459 | 51 |
| nad2 | 12,466 | 13,255 | 790 |

Supplementary Table S9 – Specimens used for mass spectrometry

Worms were collected in 2014 from eel gut, cleaned, and emptied from gonads and other internal organs.

| **Sample** | **Species** | **Number specimens** | **Comments** |
| --- | --- | --- | --- |
| HH1 | *P. laevis* | 19 | body walls, excluding proboscis |
| HH2 | *P. laevis* | 29 | body walls, excluding proboscis |
| HH3 | *P. laevis* | 39 | body walls, excluding proboscis |
| HH4 | *P. laevis* | 70 | body walls, excluding proboscis |
| HH5 | *P. laevis* | 35 | body walls, excluding proboscis |

Supplementary Table S10 – Standard InChI keys and 2D structures of candidate ligands

The ten ligands predicted to bind to the target proteins (main text Tables 1 & 2).

| Ligand | Standard InChI key and a 2D representation |
| --- | --- |
| Pranazepide | WKJDXKWFGJWGAS-XMMPIXPASA-N  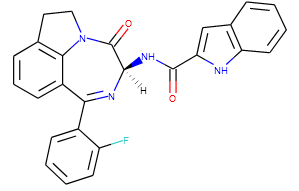 |
| Derquantel | DYVLXWPZFQQUIU-WGNDVSEMSA-N  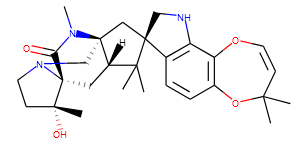 |
| Tadalafil | WOXKDUGGOYFFRN-IIBYNOLFSA-N  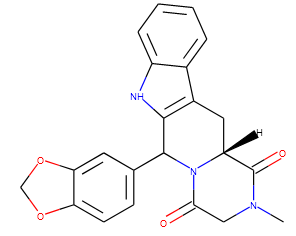 |
| Casopitant | XGGTZCKQRWXCHW-WMTVXVAQSA-N  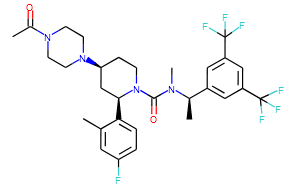 |
| Afacifenacin | IJUMFEAYOMCXAQ-UHFFFAOYSA-N  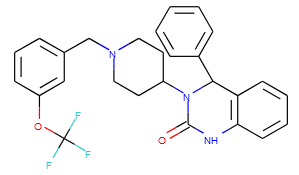 |
| Piketoprofen | ASFKKFRSMGBFRO-UHFFFAOYSA-N  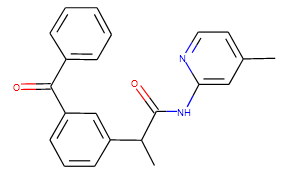 |
| Bemcentinib | KXMZDGSRSGHMMK-VWLOTQADSA-N  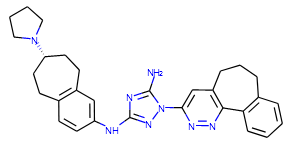 |
| Fluazuron | YOWNVPAUWYHLQX-UHFFFAOYSA-N  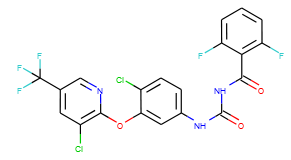 |
| Heliomycin | ABLACSIRCKEUOB-UHFFFAOYSA-N  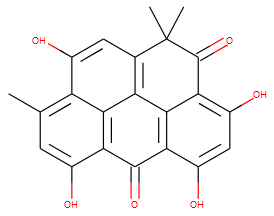 |
| Etoposide | VJJPUSNTGOMMGY-MRVIYFEKSA-N  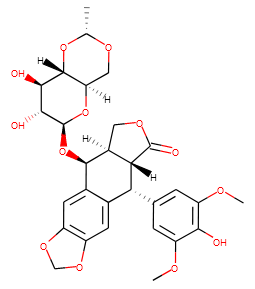 |


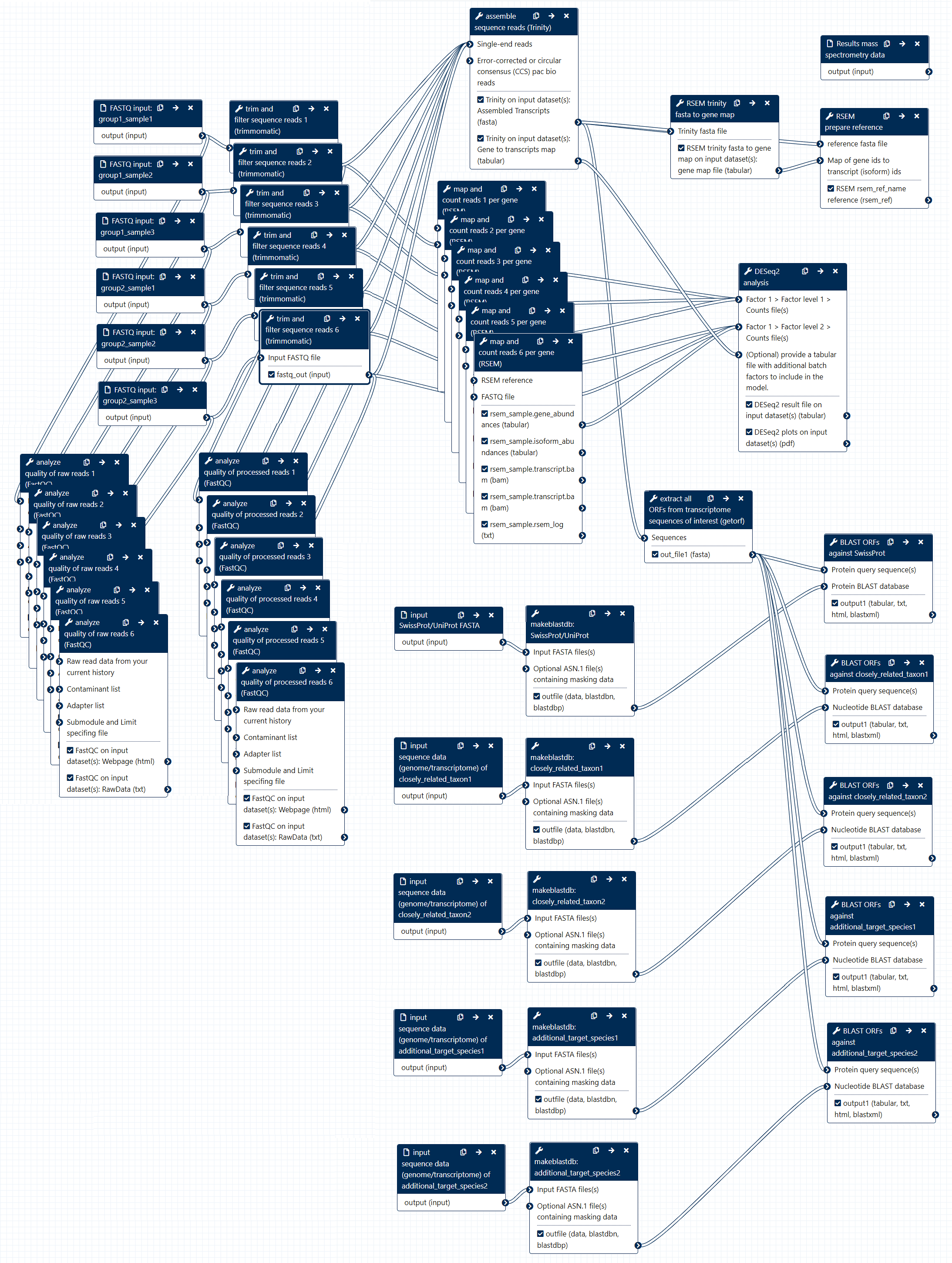


Supplementary Figure S1 – Target identification workflow at Galaxy

An example workflow for a typical study design (2 conditions, 3 replicates each) has been set up as a Galaxy workflow that can be modified based on custom needs or be used as an additional documentation resource to reproduce the pipeline in a custom UNIX system. The workflow is available at the Galaxy Europe Server: tinyurl.com/yx72rda7. See also Fig. 1.

References

[1] Bolger AM, Lohse M, Usadel B. Trimmomatic: a flexible trimmer for Illumina sequence data. Bioinformatics 2014;30:2114-20.

[2] Andrews S. FastQC: a quality control tool for high throughput sequence data; https://www.bioinformatics.babraham.ac.uk/projects/fastqc/).

[3] Bushnell B. BBMap: a fast, accurate, splice-aware aligner; https://sourceforge.net/projects/bbmap/).

[4] Yukgehnaish K, Kumar P, Sivachandran P, Marimuthu K, Arshad A, Paray BA, et al. Gut microbiota metagenomics in aquaculture: factors inﬂuencing gut microbiome and its physiological role in ﬁsh. Rev Aquacult 2020;12:1903-27.

[5] Li D, Liu C-M, Luo R, Sadakane K, Lam T-W. MEGAHIT: an ultra-fast single-node solution for large and complex metagenomics assembly via succinct de Bruijn graph. Bioinformatics 2015;31:1674-6.

[6] Simão FA, Waterhouse RM, Ioannidis P, Kriventseva EV, Zdobnov EM. BUSCO: assessing genome assembly and annotation completeness with single-copy orthologs. Bioinformatics 2015;31:3210-2.

[7] Stanke M, Keller O, Gunduz I, Hayes A, Waack S, Morgenstern B. AUGUSTUS: ab initio prediction of alternative transcripts. Nucleic Acids Res 2006;34:W435-W9.

[8] Mauer K, Hellmann SL, Groth M, Frobius AC, Zischler H, Hankeln T, et al. The genome, transcriptome, and proteome of the fish parasite *Pomphorhynchus laevis* (Acanthocephala). PLoS One 2020;15:e0232973.

[9] Hahn C, Bachmann L, Chevreux B. Reconstructing mitochondrial genomes directly from genomic next-generation sequencing reads - a baiting and iterative mapping approach. Nucleic Acids Res 2013;41:e129.

[10] Katoh K, Misawa K, Kuma Ki, Miyata T. MAFFT: a novel method for rapid multiple sequence alignment based on fast Fourier transform. Nucleic Acids Res 2002;30:3059-66.

[11] Hall TA. BioEdit: A User-friendly biological sequence alignment editor and analysis program for Windows 95/98/NT. Nucleic Acids Symposium Series 1999;41:95-8.

[12] Bernt M, Donath A, Jühling F, Externbrink F, Florentz C, Fritzsch G, et al. MITOS: Improved de novo metazoan mitochondrial genome annotation. Mol Phylogen Evol 2013;69:313-9.

[13] Meng G, Li Y, Yang C, Liu S. MitoZ: a toolkit for animal mitochondrial genome assembly, annotation and visualization. Nucleic Acids Res 2019;47:e63.

[14] Beck N, Lang B. MFannot, organelle genome annotation websever. (https://megasunbchumontrealca/cgi-bin/dev_mfa/mfannotInterfacepl 2010)

[15] Laslett D, Canbäck B. ARWEN: a program to detect tRNA genes in metazoan mitochondrial nucleotide sequences. Bioinformatics 2008;24:172-5.

[16] Wyman SK, Jansen RK, Boore JL. Automatic annotation of organellar genomes with DOGMA. Bioinformatics 2004;20:3252-5.

[17] Gissi C, Iannelli F, Pesole G. Evolution of the mitochondrial genome of Metazoa as exemplified by comparison of congeneric species. Heredity 2008;101:301-20.

[18] Kozlowski LP. Proteome-*pI*: proteome isoelectric point database. Nucleic Acids Res 2016;45:D1112-6.
